# Psychosocial interventions for intimate partner violence in low and middle income countries: A meta-analysis of randomised controlled trials

**DOI:** 10.7189/jogh.10.010409

**Published:** 2020-06

**Authors:** David T Turner, Elena Riedel, Loulou Hassan Kobeissi, Eirini Karyotaki, Claudia Garcia-Moreno, Lale Say, Pim Cuijpers

**Affiliations:** 1 Department of Clinical, Neuro and Developmental Psychology, Amsterdam Public Health Research Institute, Vrije Universiteit Amsterdam, the Netherlands; 2 UNDP/UNFPA/UNICEF/WHO/World Bank Special Programme of Research, Development and Research Training in Human Reproduction, World Health Organisation, Geneva, Switzerland

## Abstract

**Background:**

Intimate partner violence (IPV) is prevalent worldwide and presents pernicious consequences for women in developing countries or humanitarian settings. We examined the efficacy of psychosocial interventions for IPV among women in low- and middle-income countries (LMICs).

**Methods:**

Seven databases were systematically searched for randomised controlled trials (RCTs) examining psychosocial interventions for IPV in LMICs. Thirteen RCTs were included in random-effects meta-analyses. Risk ratios (RR) and risk difference were calculated as pooled effect sizes. Risk of bias was assessed using an adapted version of the Cochrane tool accounting for cluster RCTs. Sensitivity analyses were conducted for risk of bias and design characteristics. Publication bias and heterogeneity were assessed.

**Results:**

Psychosocial interventions reduced any form of IPV by 27% at shortest (relative risk (RR) = 0.73) and 25% at longest (RR = 0.75) follow up. Physical IPV was reduced by 22% at shortest (RR = 0.78) and 27% at longest (RR = 0.73) follow up. Sexual IPV was reduced by 23% at longest follow up (RR = 0.77) but showed no significant effect at shortest follow-up. Sensitivity analyses for risk of bias led to an increase in magnitude of the effect for any form of IPV and physical IPV. The effect on sexual IPV was no longer significant. Heterogeneity was moderate to high in the majority of comparisons.

**Conclusions:**

Psychosocial interventions may reduce the impact of IPV in humanitarian or low and middle income settings. We acknowledge heterogeneity and limited availability of RCTs demonstrating minimal risk of bias as limitations.

Estimates suggest that 30% of women worldwide have been subject to violence perpetrated by a partner [1]. Intimate partner violence (IPV) – including physical, sexual and emotional abuse- is a pertinent problem in low and middle income countries (LMICs) [2]. There has been increased focus on efforts to reduce IPV [3]. Sexual IPV may exacerbate the spread of HIV/AIDS and may adversely impact women by severely limiting empowerment and quality of life [4]. There are also well-established links between IPV victimisation and mental and physical health outcomes including posttraumatic stress disorder (PTSD), depression, substance abuse, chronic diseases, chronic pain and gastro-intestinal or gynaecological complications [5-8], Violence reduction efforts have also focused on post-conflict and disaster settings since humanitarian crises may exacerbate vulnerability among women in affected communities [9].

The past two decades have witnessed the development of a number of interventions attempting to reduce intimate partner violence in LMICs using psychological and/or social approaches. Primary outcome-focused examples include the targeted adaptation of established approaches such as counselling [10] or educative interventions [9]. IPV has also been increasingly included as a secondary outcome measure in RCTs targeting other outcomes related to women’s health in LMICs [11] and has been included as an outcome in RCTs implementing psychological therapy in LMICs [12]. IPV has further been a target of wider community-based interventions providing a variety of resources, often in combination with HIV prevention [13]. These *psychosocial* interventions share common characteristics in attempting to reduce IPV or related outcomes by psychological, social or educative methods.

A number of systematic reviews have investigated IPV interventions although none provide meta-analytic evidence regarding the reduction of IPV in LMICs [14-19]. Existing reviews often include quasi-experimental research and trials from high income countries. One non-meta-analytic systematic review provided preliminary findings regarding the efficacy of psychosocial intervention for IPV and primarily highlighted the necessity for better evidence on the topic [14]. We identified systematic reviews of IPV interventions in the specific populations of pregnant women and adolescents which did not focus on LMICs [15,16]. Another review focused on summarising evidence for the prevention of IPV in LMICs although included non-randomised designs [19]. A large systematic review of reviews on all forms of violence against women and girls applied the AMSTAR criteria to assess review quality although 77% of the included research- both experimental and quasi-experimental- was conducted in North America resulting in limited focus on LMICs [17]. A further publication focusing specifically on the LMIC evidence from this review concluded that there was promising evidence for interventions including community mobilisation, group training and livelihood strategies [18]. However, the inclusion in each review of non-experimental research limits the validity of findings. Since the methodology the aforementioned reviews did not allow for pooling of effect sizes, a statistical estimation of the overall impact of IPV interventions is lacking. In recent years, the availability of RCTs targeting IPV in LMICs has increased therefore we identified the need for better quality evidence in this area.

We conducted a meta-analytic review of high-quality outcome research applying psychosocial interventions to reduce IPV in LMIC or humanitarian settings. We assessed the methodological quality of the included trials to estimate the reliability of our findings and, when possible, used this information to refine our statistical estimates regarding the impact upon IPV.

## METHODS

We conducted a systematic literature search and meta-analytic review based upon PRISMA guidelines.

### Protocol

This review was as part of a broader collaborative project between the WHO Special Programme of Research, Development and Research Training in Human Reproduction in Geneva, Switzerland and the Vrije Universiteit in Amsterdam, Netherlands. A protocol for this project was registered via PROSPERO (registration number CRD42018081410).

### Search strategy

A systematic literature search was completed in November 2017 including the following databases; PubMed, Embase, PsychInfo, CINAHL, POPLINE, Global Health and Global Index Medicus. We utilised Boolean operators, MeSH terms, exploded terms and limited when possible to randomised or clinical trials. Exemplary search strings are provided in supplementary materials. Language or date restrictions were not applied. We conducted grey literature searches in databases relevant to humanitarian research including Evidenceaid, Médecins Sans Frontiéres, United Nations High Commissioner for Refugees and Forced Migration Online. Reference lists of published reviews were examined [14-19]. Search strings are included in the **Online Supplementary Document**.

### Selection of studies

We included 1) RCTs in which 2) a psychosocial intervention was compared to 3) a control condition for which 4) IPV was an outcome 5) reported by females. We included care as usual, active or attention controls, waiting-lists or no intervention as relevant comparisons. We only included trials from a) post-conflict or post-disaster circumstances (as noted in title or abstract) or b) countries defined as low to middle income based on the World Bank [20] classification system.

*Psychosocial interventions* referred to a psychological and/or social intervention which was not simply provision of material resources (eg, conditional cash transfers). We considered psychological and/or counselling-based interventions in individual [12] and group format [11], sexual and/or health education and counselling [21] and educational discussion groups [22]. We considered these *direct* forms of psychosocial intervention involving direct communication between a clinician, care provider or educator for a specified time frame. We also included community mobilisation interventions [13] and comprehensive community resource provision which included psychosocial support [23]. We considered *community intervention* a less direct form of psychosocial intervention. We determined that the term *psychosocial interventions* provided the best terminology to categorise these interventions.

RCTs in English, French, Dutch, German, Spanish and Portuguese were considered. Study selection and screening was achieved using the Covidence software (www.covidence.org) by two authors (DT and ER) who discussed all conflicts before final inclusion with consultation of a third author (EK) if necessary.

### Outcomes measures

The primary outcome measure was IPV reported by women, which we subdivided into three categories; any form of IPV, physical IPV or sexual IPV. Both continuous and dichotomous measures of IPV were considered for inclusion. The category *any form of IPV* included physical IPV, sexual IPV and non-physical forms of IPV described by RCTs as psychological, emotional or verbal. To maximise RCT availability and the power to detect effects in light of potential scarcity of RCTs, we created the *all RCTs/all IPV outcomes combined* comparison including all measures of IPV across all RCTs in a non-selective manner. We also included a category entitled *physical and/or sexual IPV* which refers to instances when physical and sexual IPV were already combined within the original RCTs since this is a regularly utilised outcome in the literature. This category did not include verbal, emotional or psychological IPV.

### Data extraction

Outcome data was extracted alongside relevant study characteristics (year of publication, date, country, setting, sample, intervention, control group and length of follow up). Due to heterogeneity in the reporting of time point measurements across RCTs (for example, one RCT may collect outcome data at six months and another after two years), we categorised outcome data either as having been assessed at a) shortest available follow up or b) longest available follow up. These categories therefore formed the basis for our meta-analytic outcome comparisons.

Data extraction was conducted and cross-checked by two authors (DT and ER) based upon guidance from the Cochrane Handbook for Systematic Reviews of Interventions [24]. Intention-to-treat data was preferred over completer data when available. Contacting authors to resolve missing data resulted in one additional inclusion [25].

### Quality assessment

In order to estimate the methodological quality of the included RCTs, we adapted the Cochrane risk of bias tool to account simultaneously for individual and cluster RCTs while providing a summary score comparable across trials. We applied five criteria of the Cochrane risk of bias assessment tool in a manner consistent with previous reviews [26,27] to provide a total score of 0-5 indicating bias risk, where 0 represents the lowest possible risk and 5 the highest. Four items of the Cochrane tool were included (sequence generation, allocation concealment, blinding of assessors and incomplete outcome data). The *other sources of bias* item addressed specific cluster RCTs issues via guidelines provided by the Cochrane collaboration [28]. Cluster RCTs were rated as “high risk” on item 5 if they violated any of 5 cluster-specific issues: 1) recruitment bias; 2) baseline imbalance; 3) loss of clusters; 4) incorrect analysis and 5) comparability with individually-randomised trials. Items were rated as high risk (1 point) or low risk (0 points). Unclear items were categorised as high risk. Two authors (DT and EK) calculated scores independently and resolved disagreements via discussion.

### Meta-analyses

The key tenet of meta-analysis is the pooling of multiple effect sizes from individual RCTs in order to provide an aggregated effect size, leading to increased statistical power and therefore more robust estimates of effect sizes in outcome research. The statistical package Comprehensive Meta-Analysis version 3 (CMA. https://www.meta-analysis.com/) was utilised for all analyses. We converted outcome data from RCTs to risk ratios (RR; a.k.a. relative risk) and calculated pooled effect sizes for each of the relevant outcome sub-categories (any, physical and sexual IPV) at each shortest and longest available follow up. In instances in which only one outcome time point was provided then this was included in both the analyses for both time points. Meta-analysis was conducted when at least five RCTs were available for each comparison category [29]. Since considerable heterogeneity was expected, a random effects model was applied in all comparisons. Forest plots were produced for each comparison to provide a visual representation of results.

Each comparison category provided an aggregated effect size reported as risk ratio. Risk difference was also reported while the number needed to treat (NNT) was also calculated to provide further insight into the utility of the interventions.

### Heterogeneity

Statistical methods estimating heterogeneity in meta-analysis aim to determine the extent to which the variation across studies may be explained by heterogeneous features of their design or the interventions tested rather than by chance. CMA provided χ^2^ tests to determine the extent of heterogeneity between included studies. The *I^2^* statistic provided an estimate of the percentage of variance arising between studies which cannot be explained by chance alone [24]. Heterogeneity may be categorised as absent, (0%), low (25%), moderate (50%) and high (75%). We calculated a 95% confidence interval for the *I^2^* statistic.

### Publication bias

Publication bias refers to the existence of the “file drawer problem” of unpublished negative RCTs artificially inflating the magnitude of the effect size. Funnel plots were examined to estimate the presence of unpublished RCTs for each comparison. Duval and Tweedie’s [30] trim and fill procedure estimated effect sizes when accounting for publication bias. Egger’s [31] test of the intercept assessed the magnitude of any publication bias and the associated statistical significance. The fail-safe N function was also utilised to estimate the number of unpublished studies that would be required for the significant effect size to drop below the 0.05 alpha level [32].

### Sensitivity analyses

We pre-specified in our protocol that sensitivity analyses would be performed in order to investigate the impact of study characteristics (eg, intervention, methodology, risk of bias) on pooled effect sizes dependent on study availability (at least 4 RCTs). Sensitivity analyses were also utilised to investigate possible sources of heterogeneity.

## RESULTS

**Figure 1:**
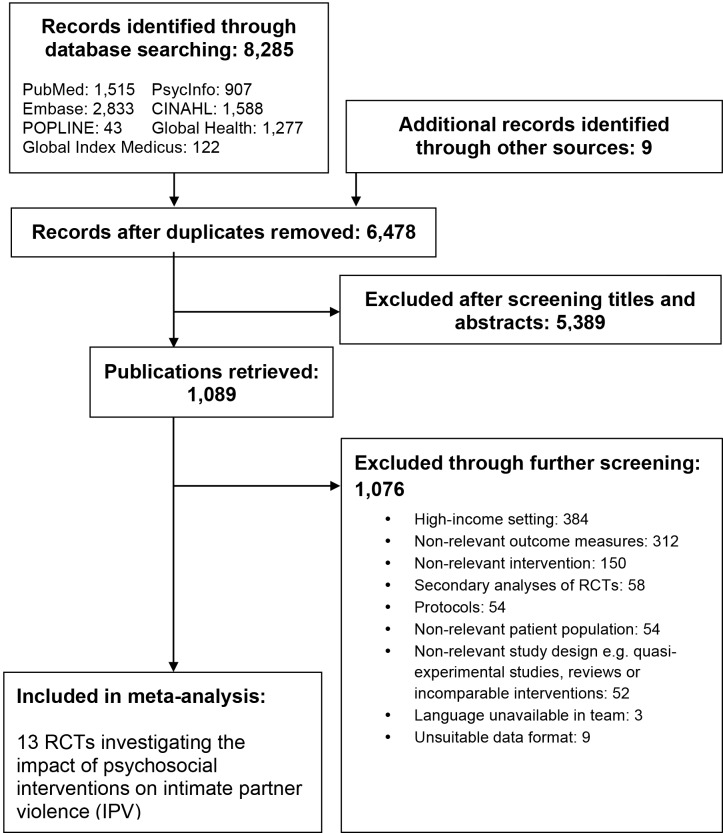
PRISMA flow diagram of the study selection process.

**Figure 1** provides the study flow diagram illustrating study inclusion. The literature search identified 8285 references, (6478 after removal of duplicates). After comparison of abstracts against inclusion and exclusion criteria, 1089 full texts were retrieved while 5389 references were excluded. Following screening of PDF full texts, 13 randomised controlled trials (RCTs) were included [9-13,21-23,25,33-36].

Study characteristics are provided in **Table 1**. We included four trials from India, three from South Africa, two from Uganda, two from Ivory Coast and one each from Pakistan and Mexico. All included RCTs were from LMICs as we did not identify any RCTs from post-conflict or post-disaster circumstances in high-income countries meeting our inclusion criteria. Interventions ranged from a short counselling and/or educative interventions ranging from 3-16 weeks (n = 9) to longer interventions ranging from 2-4 years focusing on community resource provision or “community mobilisation (n=4). The duration of one such intervention was unclear [13]. The length of follow up varied considerably.

**Table 1:** Selected characteristics of randomised controlled trials of psychosocial interventions on intimate partner violence

Study & publications	Country	Sample characteristics	Relevant comparisons & *n* in each comparison arm	IPV outcome measures	RCT Format	Bias Risk (0-4)	Duration of intervention
Abramsky 2016 [33]	Uganda	Women 18-49	Community mobilisation intervention (504) vs minimal intervention (424)	Ph & Sx, past year	Cluster	2	4 y
Gupta 2013 [22]	Ivory Coast	Women 18+	Gender dialogue group & economic empowerment (513) vs economic empowerment & wait-list (421)	Ph & Sx, past year	Cluster	3	16 weeks
Gupta 2017 [10]	Mexico	Low income women	Nurse delivered sessions focused on reduction of IPV (365) vs SC (351)	Ph & Sx, past year	Cluster	1	8 weeks
Hirani 2010 [11]	Pakistan	Low income women	Group counselling (7) vs no intervention (8)	Any, past 6 mo	Cluster	4	8 weeks
Hossain 2014 [9]	Ivory Coast	Women in post-conflict zone	Men’s IPV discussion group (113) vs SC (126)	Ph & Sx, past year	Cluster	4	16 weeks
Jewkes 2008 [21]	South Africa	Women 18+	Sexual health education and counselling (553) vs minimal intervention (550)	Any	Cluster	4	6-8 weeks
Jones 2013 [34]	South Africa	ANC women	Couples-based intervention vs minimal intervention (478) vs SC (478)	Any, past month	Cluster	1	6-8 weeks
More 2017 [23]	India	Women of reproductive age	Provision of community resource centres (7487) vs no centres (7705)	Any, past 3 mo	Cluster	1	2 y
Patel 2017 [12]	India	Depressed women 18-65	Lay counsellor brief psychological intervention (112) vs SC (120)	Ph	Individual	0	6-8 weeks
Raj 2016 [35]	India	Married women 18-30	Counselling and family planning (409) vs SC (533)	Ph & Sx, past 6 mo	Cluster	2	3 sessions
Rotheram-Borus 2015 [25]	South Africa	Pregnant women with IPV history	Home visiting intervention (543) vs SC (496)	Ph, past year	Cluster	4	2-3 y
Saggurti 2013 [36]	India	Women 18-40 with IPV history	Individual and group counselling (118) vs SC (102)	Any, past 3 mo	Cluster	4	6-9 weeks
Wagman 2015 [13]	Uganda	Women seeking HIV counselling and testing	Community mobilisation IPV intervention (1812) vs SC (2127)	Ph & Sx, past year	Cluster	2	Unclear

RCT – randomizes controlled trial, ANC – antenatal care, SC – standard care, Ph – physical IPV, Sx – sexual IPV, IPV – any form of intimate partner violence

Eight RCTs used standard care as the control, one RCT used a waiting list plus economic empowerment (which was also included in the experimental arm), two RCTs applied a minimal intervention and two trials had no intervention or no provision of resources as the control condition. The target groups for the interventions varied. Four RCTs had general inclusion of women of reproductive age (18+). Two RCTs targeted low-income women. One RCT was included for each of the following target groups; women in a post-conflict zone, women in antenatal care, depressed women, married women, pregnant women with an IPV history, women 18-40 with an IPV history and women seeking HIV counselling and testing.

**Table 2** provides the results of meta-analytic comparisons. For *any IPV*, interventions performed significantly better than controls at shortest (relative risk (RR) = 0.73, 95% confidence interval (CI) = 0.59-0.90, *P* = 0.004, *I^2^* = 80, 95% CI = 64-90*)* and longest (RR = 0.75, 95% CI = 0.60-0.94, *P* = 0.014, *I^2^* = 84, 95% CI = 72-91) follow up. This suggests a 27% and 25% lower likelihood of IPV respectively. The number needed to treat (NNT) was calculated as 16 at shortest and 18 at longest follow up.

**Table 2:** Effect sizes of psychosocial interventions on intimate partner violence

	N	RR	95% CI	*Z*	RD	NNT	*I^2^ (%)*	*I^2^* 95% CI
**Any form of intimate partner violence:‡**								
At post-treatment or shortest follow up	9	0.73**	0.59-0.90	-2.87	0.06	16	80**	64-90
At longest follow up	9	0.75*	0.60-0.94	-2.46	0.06	18	84**	72-91
**Physical intimate partner violence:**								
At post-treatment or shortest follow up	8	0.78*	0.64-0.94	-2.53	0.04	25	73**	44-87
At longest follow up	8	0.73**	0.60-0.90	-3.04	0.05	20	64**	22-83
**Sexual intimate partner violence:**								
At post-treatment or shortest follow up	6	0.90	0.75-1.08	-1.10	0.02	67	49	0-80
At longest follow up	6	0.77*	0.60-0.97	-2.19	0.04	29	53	0-66
**Physical and/or sexual IPV:‖**								
At post-treatment or shortest follow up	6	0.840	0.692-1.019	-1.774	0.04	27	54	0-80
At longest follow up	6	0.871	0.695-1.092	-1.195	0.03	33	63	0-85
**All RCTs/IPV outcomes combined: §**								
At post-treatment or shortest follow up	13	0.76**	0.63-0.92	-2.86	0.05	20	85**	76-91
At longest follow up	13	0.73**	0.56-0.89	-3.51	0.05	19	80**	66-88

IPV – intimate partner violence, RR – relative risk (risk ratio), CI – confidence interval, RD – risk difference, NNT –number needed to treat

*P < 0.05. All comparisons were using random effects model.

**P < 0.01. All comparisons were using random effects model.

‡*Any form of IPV* refers to measurement in RCTs in which any form of IPV (potentially including physical, sexual or emotional IPV) was the outcome measure.

§*All RCTs/IPV outcomes combined* refers to inclusion of these studies alongside our own combination of physical and sexual IPV in instances where the *any IPV* outcome category was not provided in the RCTs. Each of these categories may therefore include physical, sexual or emotional IPV.

‖Physical and/or sexual IPV refers to instances when physical and sexual IPV were combined within RCTs.

For *physical IPV*, interventions performed significantly better than controls at shortest (RR = 0.78, 95% CI = 0.64-0.94, *P* = 0.011, *I^2^* = 72, 95% CI = 44-87) and longest (RR = 0.73, 95% CI = 0.60-0.90, *P* = 0.002, *I^2^* = 63, 95% CI = 22-83) follow up representing a 22% and 27% reduction of IPV respectively. The number needed to treat (NNT) was estimated as 25 at shortest follow up and 20 at longest follow up.

For *sexual IPV*, interventions performed significantly better than controls at longest follow up (RR = 0.77, 95% CI = 0.60-0.97, *P* = 0.029, *I^2^* = 53, 95% CI = 0-66) representing a 23% reduction in likelihood of IPV but did not demonstrate superiority at shortest follow up (RR *=* 0.90, 95% CI = 0.75-1.08, *P* = 0.273, *I^2^* = 49, 95% CI = 0-80). The number needed to treat (NNT) was 67 at shortest and 29 at longest follow up.

For *all RCTs/IPV outcomes combined,* interventions performed significantly better than controls at shortest (RR = 0.76, 95% CI = 0.63-0.92, *P* = 0.004, *I^2^* = 84, 95% CI = 76-91) and longest (RR *=* 0.73, 95% *CI =* 0.56-0.89, *P* = 0.000, *I^2^* = 79, 95% CI = 66-88) follow up representing a 24% and 27% lower chance of IPV respectively. The number needed to treat (NNT) was calculated as 20 at shortest and 19 at longest follow up.

There was no significant difference between psychosocial interventions and controls for the combined outcome category *physical and/or sexual IPV* at shortest (RR = 0.84, 95% CI *=* 0.69-1.02, *P* = 0.076, *I^2^* = 54, 95% CI = 0-80) or longest follow up (RR = 0.87, 95% CI = 0.70-1.09, *P* = 0.232, *I^2^* = 63, 95% CI = 0-85).

Results demonstrated moderate to severe heterogeneity among all significant comparisons. Analysis of publication bias suggested that unpublished studies may exist for *any IPV* at shortest (one RCT) and longest (two RCTs) follow ups. No bias was identified for *physical IPV*, *sexual IPV* or *all RCTs/IPV*. The classic fail-safe N indicated that the number of unpublished studies required to exist to bring the effects below the significance level would be 165 (shortest follow up) and 150 (longest follow up) RCTs for the *any IPV* comparison. Egger’s [30] test of the intercept did however suggest the presence of significant publication bias (*P* < 0.05) at shortest follow up. Duval and Tweedie’s [29] trim and fill procedure removed one study; the effect of the psychosocial intervention remained significant (RR = 0.62; 95% CI = 0.58-0.67) while the magnitude of the effect increased to a 38% reduction in IPV. Egger’s test of the intercept assessed publication bias as non-significant for the longest follow up comparison. These results broadly indicate that publication bias would be unlikely to significantly alter the results.

Only one RCT (the only non-cluster RCT) [12] achieved the lowest possible risk of bias score. Six RCTs scored “high risk of bias” on 3-4 items in the risk of bias assessment therefore demonstrated considerable risk of bias. A further 5 RCT scored 1-2 items therefore demonstrated lower risk of bias. All RCTs utilised intention-to-treat analysis although in one instance high drop-out meant we judged risk of bias as still present on item 4. Only the single non-cluster RCT [12] utilised blind assessors for outcomes therefore all other RCTs had high risk of bias for blinding (item 3). Risk of bias on items 1, 2 and 5 varied across the sample. The results from the risk of bias assessment are included in **Table 3**.

**Table 3:** Adapted Cochrane Risk of Bias Tool*

Study	Item 1†	Item 2‡	Item 3§	Item 4‖	Item 5¶	Total
Abramsky et al 2016 [33]	-	-	+	-	+	2
Gupta et al 2013 [22]	+	-	+	+	-	3
Gupta et al 2017 [10]	-	-	+	-	-	1
Hirani et al 2010 [11]	+	+	+	-	+	4
Hossain et al 2014 [9]	+	+	+	-	+	4
Jewkes et al 2008 [21]	+	+	+	-	+	4
Jones et al 2013 [34]	-	-	+	-	+	1
More et al 2017 [23]	-	-	+	-	-	1
Patel et al 2017 [12]	-	-	-	-	-	0
Raj et al 2016 [35]	-	-	+	-	+	2
Rotheram-Borus et al 2015 [25]	+	+	+	-	+	4
Saggurti et al 2013 [36]	+	+	+	-	+	4
Wagman et al 2015 [13]	-	-	+	-	+	2

+ – high risk of bias; - – low risk of bias

*Total risk of bias was calculated as the sum of high risk items to provide an overall risk score. Unclear risk of bias category was disregarded therefore when no information on an item was included in the report, high risk of bias was assumed. All items were independently rated by two authors with conflicts resolved via discussion.

†Item 1, random sequence generation.

‡Item 2, allocation concealment.

§Item 3, blinding of assessors.

‖Item 4, incomplete outcome data.

¶Item 5, other risk of bias (including risk of bias due to features of cluster randomised design).

Sensitivity analysis were conducted excluding high risk of bias by including only studies with a low or moderate risk of bias (scoring 0-2 on the adapted risk of bias tool). Results are available in **Table 4**. For *any form of IPV*, it was possible to include 4 RCTs which resulted in the effect remaining significant with a slight increase in magnitude at shortest and longest follow up (RR = 0.61, 95% CI = 0.49-0.77, *P* = 0.000, *I^2^ =* 82, 95% CI = 53-93, NNT = 10) indicating a 39% reduction in IPV. Sensitivity analysis in the *physical IPV* category included 5 RCTs. The magnitude of the effect on *physical IPV* increased while remaining significant at both shortest follow up (RR = 0.73, 95% CI = 0.57-0.94, *P* = 0.014, *I^2^ =* 81, 95% CI = 57-92, NNT = 27) and longest follow up *(*RR = 0.67, 95% CI = 0.51-0.88, *P* = 0.004, *I^2^ =* 67, 95% CI = 14-87, NNT = 14) therefore indicating that psychosocial interventions reduce physical IPV by 27% at shortest follow up and 33% and longest follow up when removing RCTs with a high risk of bias.

**Table 4:** Sensitivity analyses excluding high risk of bias RCTs

	N	RR	95% CI	*Z*	RD	NNT	*I^2^ (%)*	*I^2^* 95% CI
At post-treatment or shortest follow up	4	0.61†	0.49-0.77	4.20	0.010	10	82†	53-93
**Sensitivity analyses for risk of bias**								
**Any form of intimate partner violence:**								
At post-treatment or shortest follow up	4	0.61†	0.49-0.77	4.20	0.010	10	82†	53-93
At longest follow up	4	0.67†	0.49-0.77	4.20	0.010	10	82†	53-93
Physical intimate partner violence:								
At post-treatment or shortest follow up	5	0.73†	0.57-0.94	-2.47	0.06	17	82†	57-92
At longest follow up	5	0.67†	0.51-0.88	-2.87	0.07	14	67†	14-87
**Sexual intimate partner violence:**								
At post-treatment or shortest follow up	4	0.92	0.75-1.13	0.78	0.01	100	53	0-84
At longest follow up	4	0.76	0.57-1.00	1.93	0.04	28	53	0-85
All RCTs/IPV outcomes combined:‡								
At post-treatment or shortest follow up	7	0.67†	0.53-0.87	-3.08	0.07	14	89†	83-95
At longest follow up	7	0.62†	0.52-0.74	-5.36	0.08	13	75†	48-88
**Subgroup analyses – Direct intervention**								
**Any form of intimate partner violence:**								
At post-treatment or shortest follow up	7	0.81	0.61-1.07	1.50	0.05	20	83†	67-92
At longest follow up	7	0.84	0.61-1.14	1.14	0.04	25	85†	73-93
**Physical intimate partner violence:**								
At post-treatment or shortest follow up	5	0.83*	0.69-1.00	1.97	0.04	25	0	0-79
At longest follow up	5	0.79*	0.63-0.99	2.04	0.04	25	13	0-82
**Sexual intimate partner violence:**								
At post-treatment or shortest follow up	4	0.92	0.70-1.22	0.57	0.01	100	37	0-78
At longest follow up	4	0.76	0.57-1.00	1.93	0.04	25	65*	0-88
**All RCTs/IPV outcomes combined:**								
At post-treatment or shortest follow up	9	0.78*	0.61-0.98	2.10	0.05	20	78†	60-89
At longest follow up	9	0.77*	0.59-1.00	1.97	0.05	20	81†	67-90

RCT – randomized controlled trial, CI – confidence interval, RR – relative risk (risk ratio), RD – risk difference, NNT – number needed to treat

*P < 0.05. All comparisons were using random effects model.

†*P* < 0.01. All comparisons were using random effects model.

‡*All RCTs/IPV outcomes combined* refers to inclusion of RCTs reporting *any form of IPV* alongside our own combination of physical and sexual IPV in instances where the *any IPV* outcome category was not provided in the RCTs. Each of these categories may therefore include physical, sexual or emotional IPV.

The risk of bias sensitivity analyses for *sexual IPV* included 4 RCTs. Psychosocial interventions did not demonstrate superiority at shortest or longest follow up meaning that the previously observed effect at longest follow up lost significance when controlling for high risk of bias. Sensitivity analyses were not possible for the physical and/or sexual IPV category due to limited RCT availability. Sensitivity analysis for the in *All RCTs/outcomes combined* comparison included 7 RCTs. The magnitude of the effect again increased at shortest follow up (RR = 0.67, 95% CI = 0.53-0.87, *P* = 0.002. *I^2^ =* 90, 95% CI = 83-95, NNT = 14) and longest follow up (RR = 0.62, 95% CI = 0.52-0.74, *P* = 0.000. *I^2^ =* 76, 95% CI = 48-88, NNT = 13), which represents a 33% and 38% reduction in physical IPV respectively.

After reviewing the included RCTs, we specified a sub-group of studies for sensitivity analysis consisting of RCTs focusing on *direct intervention* (primarily provision of counselling and/or education) rather than *community intervention* focusing on the provision of resources for whole communities. RCTs in the direct intervention subgroup had shorter duration of intervention (6-9 weeks) compared to the style of extended outcome measurement (2 to 4 years) after general resource provision for large geographical samples in the RCTs excluded from this subgroup.

For physical IPV, direct psychosocial interventions performed better than controls at shortest follow up (RR = 0.83, 95% CI = 0.69-1.00, *P* = 0.049. *I^2^ = 0,* 95% CI = 0-79, NNT = 25) and at longest follow up (RR = 0.79, 95% CI = 0.63-0.99, *P* = 0.042. *I^2^ =* 13, 95% CI = 0-82, NNT = 25) representing a 17% and 21% reduction in IPV respectively. When all RCTs and outcomes were included, direct psychosocial interventions performed better than controls at shortest (RR = 0.78, 95% CI = 0.61-0.98, *P* = 0.04. *I^2^ =* 78, 95% CI = 60-89, NNT = 20) and longest (RR = 0.77, 95% CI = 0.59-1.00, *P* = 0.04. *I^2^ =* 81, 95% CI = 67-90, NNT = 20) follow up with a 22% and 23% reduction in IPV respectively. Psychosocial interventions did not demonstrate superiority over controls for *any form of IPV* or *sexual IPV*.

## DISCUSSION

This review is, to our knowledge, the first attempt to systematically pool effect sizes from the developing evidence base for psychosocial interventions attempting to reduce IPV in LMICs and/or humanitarian settings. Interventions were broadly effective in reducing IPV perpetuated against women in these communities. We estimated that intervention reduces IPV by up to 27% for any form of IPV, up to 27% for physical IPV and up to 23% for sexual IPV. The finding for sexual IPV was less robust since we only demonstrated effects at longest but not shortest follow up. We note however that this category was the most underpowered and risked type-II error.

In sensitivity analyses removing high risk of bias, the magnitude of effects increased. Psychosocial interventions reduced IPV by up to 39% for any form of IPV and up to 38% for physical IPV. This pattern of increasing magnitude as we removed lower quality research adds to the robustness of our findings.

Since comparisons for sexual IPV were non-significant when excluding RCTs with a high risk of bias, it is possible that psychosocial interventions are more reliably efficacious in reducing physical than sexual IPV. The sensitivity analysis for sexual IPV and *any form of IPV* suffered from minimal RCT availability and therefore limited power to detect effects [32]. This limitation, alongside the finding that psychosocial interventions were superior to control conditions at longest follow-up, means that it is too early to rule out broader efficacy of psychosocial interventions targeting sexual IPV. We also note that the reduction of sexual IPV was not the primary outcome target of all RCTs in which it was examined. It is possible that the impact of psychosocial intervention on sexual IPV is more protracted therefore difference between groups was only observed at longest follow up. However, this remains a question of relevance for future research and may become clearer as more high-quality RCTs become available. We also note the non-significant finding in the *physical and/or sexual IPV* category although acknowledge that this comparison contained only a subset of available RCTs and does not therefore provide the most comprehensive available evidence.

This review has a number of strengths. Including only RCTs allows a clearer estimate of the true effects of an intervention and limits the ‘noise’ which hinders reviews in this field. A further strength is that we assessed risk of bias based on the specific methodology of (cluster) RCTs and applied sensitivity analyses demonstrating that RCTs with higher methodological stringency produced results more strongly in favour of psychosocial interventions.

An important limitation is the existence of moderate to severe heterogeneity across comparisons. While the IPV outcomes were relatively heterogeneous, we included a broad range of psychosocial interventions ranging from individual counselling to community-wide, interventions assessed at a population level. IPV was in some instances the primary outcome but in others a secondary outcome for which the intervention was not specifically designed. We note that the comparison which concluded that direct forms of psychosocial intervention were superior to controls for physical IPV was the only comparison displaying minimal heterogeneity. This suggests that comparing direct rather than community interventions provided the most homogenous comparison but unfortunately low study availability limits firmer conclusions in this area.

Similarly, we included one individual RCTs alongside cluster RCTs of widely varying scale, design and methodology. There was also heterogeneity in the target population across RCTs; eg, Patel et al (2017) [12] focused solely on depressed women while Rotheram-Borus et al (2015) [25] focused on pregnant women with history of IPV. There was also considerable variability in the components and approaches subsumed in our “psychosocial interventions” umbrella terminology. Combining disparate interventions, populations and designs risks comparing “apples with oranges” in a form of potentially ‘forced harmonisation’ and may limit definitive comment on which interventions are appropriate and applicable. Such factors may also explain high levels of heterogeneity. Due to limited study availability, it was also necessary to include IPV measured across any time frame within our meta-analytic comparisons rather than specific, homogenous measurements (eg, IPV reported in the past 6 months). This limitation may also introduce heterogeneity. We also note the likelihood that the stigma and social unacceptability associated with experiencing and reporting IPV may have an impact on the validity of outcome measurement, especially in patriarchal cultures.

Despite these limitations, it is reasonable to suggest that our review demonstrates at the very least that violence perpetrated by partners against women in LMICs is a worthy target for psychosocial intervention and that there is the potential for considerable reduction of its impact; up to 38% reduction for all forms of IPV combined among higher-quality RCTs is a remarkable finding. This category contained 7 RCTs consisting of three community mobilisation interventions and four interventions focused on counselling and/or psychological intervention and education, potentially indicating that both approaches are efficacious in combating IPV. Our results suggest that the reduction of IPV via psychosocial means is achievable and adds validity justify the application of such intervention methods.

A further limitation inherent to meta-analysis is that we were unable to comment on the efficacy of specific formats of intervention or their components. There was limited availability of specific sub-types of psychosocial intervention meaning we lacked sufficient power to compare various forms of psychosocial intervention in a meaningful manner. In order to investigate the effect of intervention components, complex dismantling studies would be required in order to understand their relative contribution. Such studies are rare even for well-established psychological interventions therefore represent a later developmental stage of the evidence base. We were able to perform a sub-group analysis at the study level including only trials with “direct” intervention. However, it remains an important consideration that future research may further dismantle and distil the effective elements of psychosocial interventions in this field since there remains limited understanding of the effective elements. Similarly, further high-quality RCTs which continue to specify sub-categories such as physical and sexual IPV will aid the development of future meta-analytic reviews while allowing better comparison of sub-categories of psychosocial interventions. In order to broadly facilitate the next developmental steps of outcome research in this field, the development of harmonised RCTs with more standardised, consensus-based approaches toward outcome monitoring and intervention delivery across trials may improve their comparability. This may include clearer labelling or “branding” of interventions in a manner comparable to mainstream psychological intervention research (for example “cognitive behavioural therapy” or “acceptance and commitment therapy”) and consensus on the most important time points and categories for IPV outcome measurement thus reducing heterogeneity. Wider inclusion of IPV as a primary outcome is also important since many trials included IPV in a long list of secondary outcome measures. This may in turn be indicative of limited attention or resources directed toward supporting accurate reporting of IPV despite stigma.

This review provides clear evidence for the efficacy of psychosocial interventions for IPV. Further high-quality outcome research is required to tease apart the problems of heterogeneity and RCT availability to investigate more “distilled” subgroups of psychosocial interventions.

On a meta-analytical level it is too early to specify exactly which elements and styles of psychosocial intervention are most effective in reducing violence toward women, but our evidence suggests that there is value in the ongoing application of varied psychological intervention packages to help protect women in LMICs. Despite the noted limitations, we regard our evidence as clear recommendation that efforts to further develop and implement IPV interventions in affected communities continue and receive adequate resource provision. Our findings provide the most comprehensive available evidence to date that provision of psychosocial intervention can protect many women in LMICs from the impact of IPV, therefore it is important that we capitalise upon this possibility. We note that the vast majority of eligible RCTs were conducted in sub-Saharan Africa or South Asia, which although demonstrative of progress in these areas emphasises that research and implementation in LMICs is highly limited in large regions globally thus leaving significant room for expansion. Our findings therefore have considerable implication for the delivery of intervention packages to vulnerable and disadvantaged communities worldwide. We recognise the potential that such interventions contribute broadly to the reduction of adverse outcomes for women and families in LMICs including trauma, psychopathology, economic disadvantage, health inequalities and communicable diseases.

## Additional material

Online Supplementary Document
