# Supplementary material for: Psychosocial interventions for intimate partner violence in low and middle income countries: A meta-analysis of randomised controlled trials
Source: J Glob Health. 2020 Apr 7;10(1):010409. doi: 10.7189/jogh.10.010409 (PMC7182699; doi:10.7189/jogh.10.010409)
Supplement: Online Supplementary Document [file jogh-10-010409-s001.pdf]

Supplementary Materials

Figures S1-S8. Forest plots from meta-analytic comparisons.

Any IPV at shortest follow-up

Study name	Risk ratio	Lower limit	Upper limit	Z-Value	p-Value
Abramsky 2016	0,616	0,494	0,768	-4,310	0,000
Gupta 2013	0,985	0,757	1,281	-0,114	0,909
Gupta 2017	0,964	0,720	1,291	-0,243	0,808
Hirani 2010	0,914	0,396	2,110	-0,210	0,834
Hossain 2014	0,659	0,349	1,246	-1,284	0,199
Jewkes 2008	0,899	0,708	1,141	-0,877	0,380
Jones 2013	0,473	0,378	0,591	-6,591	0,000
More 2017	0,544	0,493	0,600	-12,206	0,000
Saggurti 2013	1,017	0,564	1,834	0,056	0,955
	0,730	0,589	0,905	-2,872	0,004

Statistics for each study

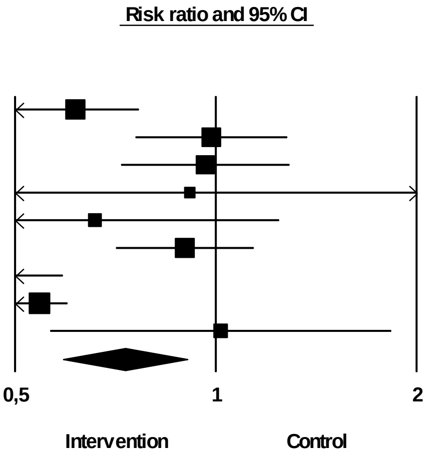

Any IPV at longest follow-up

Study name	Risk ratio	Lower limit	Upper limit	Z-Value	p-Value
	Statistics for each study				
Abramsky 2016	0,616	0,494	0,768	-4,310	0,000
Gupta 2013	0,985	0,757	1,281	-0,114	0,909
Gupta 2017	0,964	0,720	1,291	-0,243	0,808
Hirani 2010	0,914	0,396	2,110	-0,210	0,834
Hossain 2014	0,659	0,349	1,246	-1,284	0,199
Jewkes 2008	1,087	0,811	1,459	0,559	0,576
Jones 2013	0,473	0,378	0,591	-6,591	0,000
More 2017	0,544	0,493	0,600	-12,206	0,000
Saggurti 2013	1,017	0,564	1,834	0,056	0,955
	0,749	0,595	0,943	-2,455	0,014

Risk ratio and 95% CI

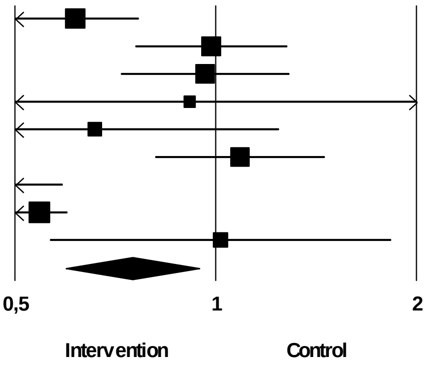

Physical IPV at shortest follow-up

Study name	Risk ratio	Lower limit	Upper limit	Z-Value	p-Value
	Statistics for each study				
Abramsky 2016	0,416	0,300	0,578	-5,226	0,000
Gupta 2013	0,740	0,520	1,053	-1,674	0,094
Gupta 2017	0,957	0,846	1,083	-0,698	0,485
Hossain 2014	1,115	0,459	2,711	0,240	0,810
Patel 2017	0,536	0,290	0,990	-1,993	0,046
Raj 2016	0,754	0,562	1,012	-1,878	0,060
Wagman 2015	0,921	0,807	1,051	-1,224	0,221
Rotheram-Borus 2015	1,012	0,758	1,352	0,082	0,935
	0,783	0,649	0,946	-2,535	0,011

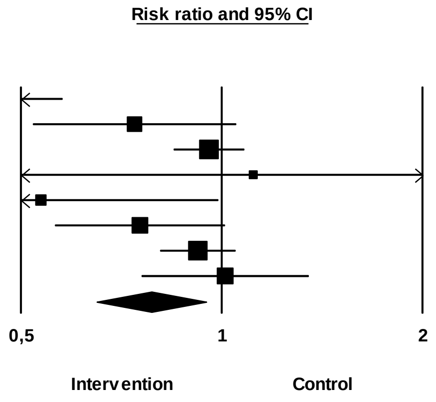

Physical IPV at longest follow-up

Study name	Statistics for each study				
	Risk ratio	Lower limit	Upper limit	Z-Value	p-Value
Abramsky 2016	0,416	0,300	0,578	-5,226	0,000
Gupta 2013	0,740	0,520	1,053	-1,674	0,094
Gupta 2017	0,957	0,846	1,083	-0,698	0,485
Hossain 2014	1,115	0,459	2,711	0,240	0,810
Patel 2017	0,536	0,290	0,990	-1,993	0,046
Raj 2016	0,681	0,505	0,918	-2,524	0,012
Wagman 2015	0,736	0,629	0,862	-3,806	0,000
Rotheram-Borus 2015	0,947	0,700	1,282	-0,351	0,726
	0,731	0,598	0,895	-3,036	0,002

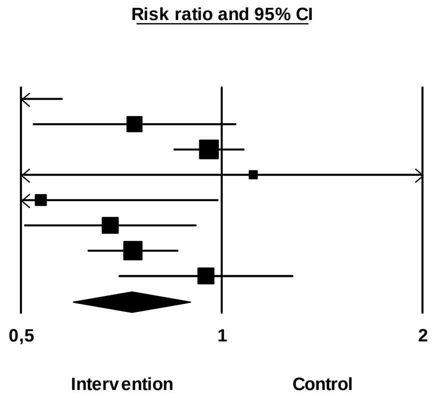

Sexual IPV at shortest follow-up

Study name	Risk ratio	Lower limit	Upper limit	Z-Value	p-Value
Abramsky 2016	0,699	0,524	0,934	-2,419	0,016
Gupta 2013	0,986	0,707	1,374	-0,086	0,931
Gupta 2017	1,116	0,862	1,445	0,832	0,405
Hossain 2014	0,438	0,190	1,008	-1,940	0,052
Raj 2016	0,790	0,460	1,356	-0,856	0,392
Wagman 2015	1,005	0,864	1,168	0,061	0,951
	0,903	0,751	1,084	-1,095	0,273

Risk ratio and 95% CI

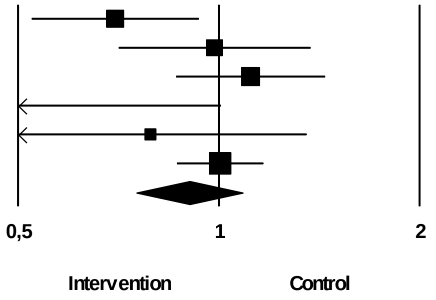

Sexual IPV at longest follow-up

Study name	Risk ratio	Lower limit	Upper limit	Z-Value	p-Value
Abramsky 2016	0,699	0,524	0,934	-2,419	0,016
Gupta 2013	0,986	0,707	1,374	-0,086	0,931
Gupta 2017	1,116	0,862	1,445	0,832	0,405
Hossain 2014	0,438	0,190	1,008	-1,940	0,052
Raj 2016	0,466	0,287	0,756	-3,096	0,002
Wagman 2015	0,751	0,625	0,902	-3,065	0,002
	0,767	0,604	0,973	-2,186	0,029

Statistics for each study

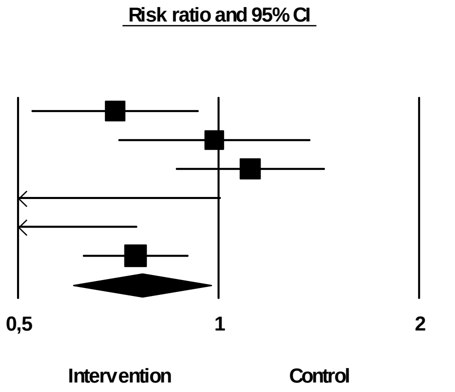

All RCTs and IPV outcomes combined at shortest follow-up

Study name	Outcome	Risk ratio	Lower limit	Upper limit	Z-Value	p-Value
		**Statistics for each study**				
Abramsky 2016	Any IPV	0,616	0,494	0,768	-4,310	0,000
Gupta 2013	Any IPV	0,985	0,757	1,281	-0,114	0,909
Gupta 2017	Any IPV	0,964	0,720	1,291	-0,243	0,808
Hirani 2010	Any IPV	0,914	0,396	2,110	-0,210	0,834
Hossain 2014	Any IPV	0,659	0,349	1,246	-1,284	0,199
Jewkes 2008	Any IPV	0,899	0,708	1,141	-0,877	0,380
Jones 2013	Any IPV	0,473	0,378	0,591	-6,591	0,000
More 2017	Any IPV	0,544	0,493	0,600	-12,206	0,000
Patel 2017	Physical	0,536	0,290	0,990	-1,993	0,046
Raj 2016	Combined	0,772	0,499	1,192	-1,167	0,243
Saggurti 2013	Any IPV	1,017	0,564	1,834	0,056	0,955
Wagman 2015	Combined	0,962	0,835	1,108	-0,537	0,591
		0,745	0,611	0,908	-2,919	0,004

Risk ratio and 95% CI

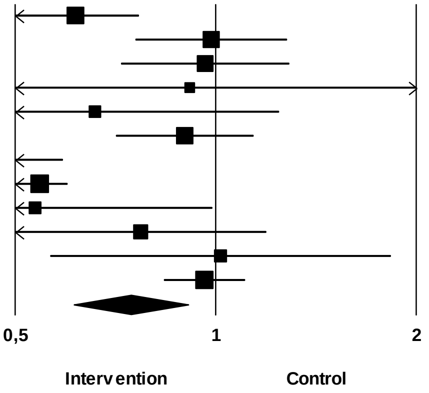

Physical IPV at longest follow-up

Study name	Risk ratio	Lower limit	Upper limit	Z-Value	p-Value
	**Statistics for each study**				
Abramsky 2016	0,416	0,300	0,578	-5,226	0,000
Gupta 2013	0,740	0,520	1,053	-1,674	0,094
Gupta 2017	0,957	0,846	1,083	-0,698	0,485
Hossain 2014	1,115	0,459	2,711	0,240	0,810
Patel 2017	0,536	0,290	0,990	-1,993	0,046
Raj 2016	0,681	0,505	0,918	-2,524	0,012
Wagman 2015	0,736	0,629	0,862	-3,806	0,000
Rotheram-Borus 2015	0,947	0,700	1,282	-0,351	0,726
	0,731	0,598	0,895	-3,036	0,002

Risk ratio and 95% CI

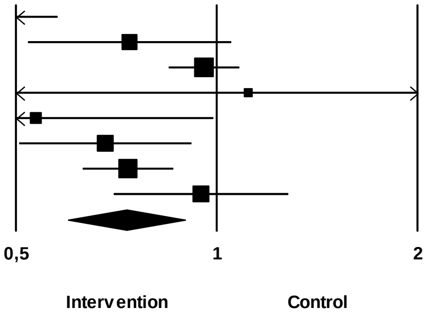

Appendix S1. Exemplary search string used in PubMed.

Psychotherapy [MH] OR psychotherap* [All Fields] OR “psychological therapy” [All Fields] OR counseling [MH] or counsel* [All Fields] OR “supportive counseling” [All Fields] OR “supportive therapy” [All Fields] OR social support [MH] OR “social support” [All Fields] OR “support work” [All Fields] OR sex counseling [MH] OR sex counsel* [All Fields] OR “sex therapy” [All Fields] OR sex education [MH] OR “sex education [All Fields] OR “preventive care” [All Fields] OR community mental health services [MH] “community mental health” [All Fields] OR health education [MH] “health education [All Fields] OR “community intervention” [All Fields] OR “community support” [All Fields] OR “community education” [All Fields] OR “empowerment programme” [All Fields] OR “psychosocial intervention” [All Fields] OR “reproductive healthcare” [All Fields] OR “education campaign” [All Fields] OR prenatal care [MH] OR “prenatal care” [All Fields] OR prenatal education [MH] OR “prenatal education” [All Fields] OR “prenatal intervention” [All Fields] OR “prenatal support” [All Fields] OR perinatal care [MH] OR “perinatal care” [All Fields] OR “perinatal intervention” [All Fields] OR “perinatal support” [All Fields]

AND (enter in next box in advanced search option)

reproductive health [MH] OR reproductive medicine [MH] OR “sexual health” [All Fields] OR intimate partner violence [MH] OR “intimate partner violence” [All Fields] OR “gender-based violence” [All Fields] OR “gender based violence” [All Fields] OR sexual violence [MH] OR “sexual violence” [All Fields] OR rape [MH] OR “rape” [All Fields] OR domestic violence [MH] OR “domestic violence” [All Fields] OR “domestic abuse” [All Fields] OR sexual abuse [MH] OR “sexual abuse” [All Fields] OR maternal health [MH] OR “maternal health” [All Fields] OR perinatal mortality [MH] OR “perinatal mortality” [All Fields] OR perinatal death [MH] OR “perinatal death” [All Fields] OR “newborn death” [All Fields] OR “new-born death” [All Fields] OR “new born death” [All Fields] OR fetal death [MH] OR “fetal death” [All Fields] OR stillbirth [MH] OR “stillbirth” [All Fields] OR “still birth” [All Fields] OR fertility [MH] or fertility [All Fields] OR fertility preservation [MH] OR “fertility preservation” [All Fields] OR female infertility [MH] OR “female infertility” [All Fields] OR psychological sexual dysfunction [MH] OR physiological sexual dysfunction [MH] OR libido [MH] OR sexual behaviour [MH] OR sexual partners [MH] OR sexuality [MH] OR “sexuality” [All Fields] OR contraception [MH] OR “contraception” [All Fields] OR “contracepti*” [All Fields] OR “contraceptive use” [All Fields] OR condoms [MH] OR “condom*” [All Fields] OR “condom use” [All Fields] OR Reproductive Health Services [MH] OR “Reproductive Health Services” [All Fields] OR “Reproductive plan*” [All Fields] OR “Postcoital Contraception”[MH] OR “post-coital contraception” [All Fields] OR Immunologic Contraception [MH] OR “immunologic contraception” [All Fields] OR Barrier Contraception [MH] OR Contraception Behaviour [MH] OR “contracep* behavior” [All Fields] OR “birth control” [All Fields] OR “birth spacing” [All Fields] OR “child spacing” [All Fields] OR “the pill” [All Fields] OR “oral contraceptive” [All Fields] OR “microbicide” [All Fields] OR “diaphragm” [All Fields] OR “IUD” [All Fields] OR “Intrauterine device” [All Fields] OR Medicated Intrauterine Devices [MH] OR Copper Intrauterine Devices [MH] OR Intrauterine Devices [MH] OR “Drug implant*” [MH] OR Drug implant* [MH] OR “Drug Pellet*” [All Fields] OR Levonorgestrel [MH] OR Norethindrone [MH] OR “contraceptive implant*” [All Fields] OR “progestogen only contraceptive*” [All Fields] OR “progestogen implant*” [All Fields] OR “etonogestrel implants” [All Fields] OR “Implanon” [All Fields] OR “Subdermal contraceptive implant*” [All Fields] OR “Norplant” [All Fields] OR “Jadelle” [All Fields] OR “Sino-implant” [All Fields] OR “Depo Provera” [All Fields] OR “Nexplanon” [All Fields] OR “Norprogesterones” [All Fields] OR “natural family planning” [All Fields] OR “lactational amenorrhea” [All Fields] OR “LAM” [All Fields] OR “postpartum amenorrhea” [All Fields] OR “post-partum amenorrhea” [All Fields] OR “amenorrhea” [MH] OR “periodic abstinence” [All Fields] OR “rhythm method” [All Fields] OR “calendar method” [All Fields] OR “sexual abstinence” [All Fields] OR Family Planning Services [MH] OR Natural Family Planning Methods [MH] OR Female Contraceptive Devices [MH] OR Female Contraceptive Agents [MH] OR Female Condoms [MH] OR Reproductive Sterilization [MH] OR “family planning” [All Fields] OR population control [MH] OR Induced abortion [MH] OR abortion [All Fields] OR Incomplete Abortion [MH] OR Spontaneous Abortion [MH] OR “abortion” [All Fields] OR “miscarriage” [All Fields] OR “pregnancy termination” [All Fields] OR “termination of pregnancy” [All Fields] OR “abortal" [All Fields] OR "postabortion" [All Fields] OR "post-abortal"[All Fields] OR “postabortion care” [All Fields] OR “incomplete abortion*” [All Fields] OR "Mifepristone" [MH] OR "Misoprostol" [MH] OR “RU486” [All Fields] OR “mifegyne” [All Fields] OR “Cytotec” [All Fields] OR “Medabon” [All Fields] OR “medication abortion” [All Fields] OR “medical abortion” [All Fields] OR "unsafe abortion" [All Fields] OR "unsafe abortions" [All Fields] OR "aftercare" [All Fields] OR "after care" [All Fields] OR Obstetrical Extraction [MH] OR "Vacuum Curettage" [MH] OR “surgical abortion” [All Fields] OR “D&E”

[All Fields] OR “suction curettage” [All Fields] OR “vacuum aspiration” [All Fields] OR “D&C” [All Fields] OR “menstrual regulation” [All Fields] OR Postoperative Hemorrhage [MH] OR Uterine Hemorrhage [MH] OR Postpartum Hemorrhage [MH] OR Infection [MH] OR Pelvic Infection [MH] OR Uterine Perforation [MH] OR Uterine Rupture [MH] OR Pregnancy Complications [MH] OR Postoperative Complications [MH] OR Intraoperative Complications [MH] OR Emergency Treatment [MH] OR Septic Abortion [MH] OR haemorrhage [MH] OR “haemorrhage” [All Fields] OR “hemorrhage” [All Fields] OR “bleeding” [All Fields] OR “endometritis” [All Fields] OR “parametritis” [All Fields] OR “metritis” [All Fields] OR “pelvic infection” [All Fields] OR “uterine infection” [All Fields] OR “uterine perforation” [All Fields] OR “abortion-related complications” [All Fields] OR “emergency care” [All Fields] OR “ongoing pregnancy” [All Fields] OR “ectopic pregnancy” [All Fields] OR “emergency treatment” [All Fields] OR “EmOC” [All Fields] OR “emergency obstetric care” [All Fields] OR “complications” [All Fields] OR “stillb*” [All Fields] OR “birth” [All Fields] OR HIV [MH] OR “human immunodeficiency syndrome” [All Fields] OR acquired immunodeficiency syndrome [MH] OR “acquired immunodeficiency syndrome” [All Fields] OR “AIDS” [All Fields] OR “HIV/AIDS” [All Fields] OR “Human Immunodeficiency Virus” [MH] OR “Human Immune Deficiency Virus” [All Fields] OR HIV-1 [MH] OR HIV-2 [MH] OR “HIV infections” [MH] OR “HIV seropositivity” [MH] OR “HIV seroprevalence” [MH] OR “AIDS serodiagnosis” [MH] OR sexually transmitted disease$ [MH] OR “sexually transmitted” [All Fields] OR “STDs” [All Fields] OR “STIs” [All Fields] OR Chlamydia infections [MH] OR Gonorrhea [MH] OR “chlamydia” [All Fields] OR “gonorrhoea” [All Fields] OR “syphilis” [All Fields] OR “syphilis” [All Fields] OR “sexually transmitted infections”[MH] OR “sexually transmitted infection$” [All Fields] OR “sexually transmitted disease$” [All Fields] OR “hepatitis” [All Fields] OR “chancroid” [All Fields] OR “trichomoniasis” [All Fields] OR “human papillomavirus” [All Fields] OR “HPV” [All Fields] OR “genital wart$” [All Fields] OR “herpes” [All Fields] OR “bacterial vaginosis” [All Fields] OR “scabies” [All Fields] OR “public lice” [All Fields] OR “crab lice” [All Fields] OR “pelvic inflammatory disease” [All Fields] OR “PID” [All Fields] OR “mucopurulent cervicitis” [All Fields] OR “MPC” [All Fields] OR “molluscum contagiosum” [All Fields] OR “lymphogranuloma venereum” [All Fields] OR “LGV” [All Fields] OR “antenatal HIV test” [All Fields] OR “maternal HIV test” [All Fields] OR “infant HIV test” [All Fields] OR “infant HIV diagnosis” [All Fields] OR “option A” [All Fields] OR “option B” [All Fields] OR “option B+” [All Fields] OR “nevirapine” [All Fields] OR “mother-to-child transmission” [All Fields] OR maternal transmission [MH] “maternal transmission” [All Fields] OR MTCT [All Fields] OR “prevention of mother-to-child transmission” [All Fields] OR “PMTCT” [All Fields] OR “PMTCT cascade” [All Fields] Pregnan* [MH] OR Unplanned Pregnancy [MH] OR unwanted pregnancy [MH] OR “Pregnancy in adolescence”[MH] OR “Pregnancy outcome”[MH] OR “Pregnan*”[All Fields] OR “IUP” [All Fields] OR “Intrauterine pregnancy”[All Fields] OR “Maternal health” [MH] OR “Maternal health” [All Fields] OR “Maternal welfare” [MH] OR “Maternal welfare” [All Fields]OR “Safe motherhood”[MH] OR “Safe motherhood”[All Fields] OR “Perinatal”[MH] OR “Perinatal”[All Fields] OR “Perinatal care”[MH] OR “Perinatal health”[MH] OR “Prenatal care”[MH] OR “Prenatal health”[MH] OR “Prenatal diagnosis”[MH] OR “Perinatal care”[All Fields] OR “Perinatal health”[All Fields] OR “Postnatal health”[MH] OR “Antenat*”[MH] OR “Antenatal health”[MH] OR “Antenatal*”[All Fields] OR “Ante-natal*”[All Fields] OR “Prenatal*”[MH] OR “Prenatal*”[All Fields] OR “postnatal care”[All Fields] OR “postnatal”[All Fields] OR “post-natal”[All Fields] OR “Postpart*”[MH] OR “Post-part*” [MH] OR “Parturition”[MH] OR “Postpartum period”[MH] OR “Postpartum”[All Fields] OR “Post-partum”[All Fields] OR “Puerperium”[All Fields] OR “Gestation”[All Fields] OR “postbirth”[All Fields] OR “Post-birth” [All Fields] OR “Mother*” [MH] OR “Matern*” [MH] OR “Childbirth” [All Fields] OR “Obstetrics”[All Fields] OR “obstetric*” [MH] OR “Gynecology” [MH] OR “Labor pain” [MH] OR “Obstetric Surgical Procedures” [MH] OR “Safe delivery” [MH] OR “Safe delivery” [All Fields] OR “Skilled birth attend*” [MH] OR “Skilled birth attend*” [All Fields] OR “Maternal Health Service*” [MH] OR “Emergency Obstetric Care” [All Fields] OR “EmOC” [All Fields] OR “EmONC” [All Fields] OR “Infant” [MH] OR “Neonat*” [MH] OR “Neonatal health” [MH] OR “Infant health” [MH] OR “Newborn health” [MH] OR “Newborn infant health” [MH] OR “Infant welfare” [MH] OR “Baby health” [MH] OR “Newborn” [MH] OR “Fetus” [MH] OR “Fetal therapies” [MH] OR “Fetal monitoring” [MH] OR “Infant” [All Fields] OR “Neonat*” [All Fields] OR “Neonatal health” [All Fields] OR “Infant health” [All Fields] OR “newborn health” [All Fields] OR “Newborn infant health” [All Fields] OR “Infant welfare” [All Fields] OR “Baby health” [All Fields] OR “Newborn” [All Fields] OR “Stillbirth” [MH] OR “Stillbirth” [All Fields] OR “Still-birth” [All Fields] OR “adolescent sexual health” [All Fields] OR “adolescent reproductive health” [All Fields] OR “adolescent health” [All Fields] OR “adolescent health services” [All Fields] OR “youth friendly services” [All Fields] OR “adolescent friendly services” [All Fields] OR “adolescent health service” [All Fields] OR “youth friendly service” [All Fields] OR “adolescent friendly service” [All Fields] OR “youth program*” [All Fields] OR “gender-based violence” [All Fields] OR “partner violence”[All Fields] OR “family violence” [All Fields] OR “violence against women” [All Fields] OR “domestic violence” [All Fields] OR “sexual abuse” [MH]

OR “sexual abuse” [All Fields] OR “sex crime” [All Fields] OR “sexual crime” [All Fields] OR “domestic violence” [All Fields] OR “domestic violence” [MH] OR “family violence” [MH] or “sexual violence” [All Fields] OR “sexual violence” [MH] OR “rape” [All Fields] OR “physical violence” [All Fields] OR “rape” [MH] OR “intimate partner violence” [All Fields] OR “intimate partner violence” [MH] OR “partner violence” [All Fields] OR “partner abuse” [All Fields] OR “spousal abuse” [All Fields] OR “spouse abuse”[MH] OR “wife abuse” [All Fields] OR “partner violence” [All Fields] OR “partner violence”[MH] OR “assault” [All Fields] OR “physical assault” [All Fields] OR “sexual assault” [All Fields] OR “sexual crime” [All Fields] OR “sexual harassment” [MH] OR “sexual harassment” [All Fields] OR “sexual coercion” [All Fields] OR “forced sex” [All Fields] OR “sexual slavery” [All Fields] OR “abused woman” [All Fields] OR “abused women” [All Fields] OR “battered woman” [All Fields] OR “battered women” [All Fields] OR “battered women” [MH] OR "postoperative" [All Fields] OR "post-operative" [All Fields] OR Hemorrhage [MH] OR victimisation [All Fields] OR victim* [All Fields]
